# Moving Immunoprevention Beyond Virally Mediated Malignancies: Do We Need to Link It to Early Detection?

**DOI:** 10.3389/fimmu.2019.02385

**Published:** 2019-10-10

**Authors:** Madhav V. Dhodapkar, Kavita M. Dhodapkar

**Affiliations:** ^1^Department of Hematology and Medical Oncology, Emory University, Atlanta, GA, United States; ^2^Winship Cancer Institute, Emory University, Atlanta, GA, United States; ^3^Department of Pediatrics, Aflac Cancer and Blood Disorders Center of Children's Healthcare of Atlanta, School of Medicine, Emory University, Atlanta, GA, United States

**Keywords:** preneoplasia, early detection, cancer prevention, T cell exhaustion, cancer vaccine, cancer interception

## Abstract

Vaccines can successfully prevent viral infections and have emerged as an effective strategy for preventing some virally mediated malignancies. They also represent our major hope for cost-effective reduction of the cancer burden. The concept that the immune system mediates surveillance and editing roles against tumors is now well-established in murine models. However, harnessing the immune system to prevent human cancers that do not have a known viral etiology has not yet been realized. Most human cancers originate in a premalignant phase that is more common than the cancer itself. Many of the genetic changes that underlie carcinogenesis originate at this stage when the malignant phenotype is not manifest. Studies evaluating host response in human premalignancy have documented that these lesions are immunogenic, setting the stage for immune-based approaches for targeted prevention of human cancer. However, recent studies suggest that the hierarchy of T cell exhaustion and immune-suppressive factors have already begun to emerge in many preneoplastic states. These considerations underscore the need to link immune prevention to earlier detection of such lesions and to personalize such approaches based on the status of the pre-existing immune response.

## Why Prevention?—Lessons From Virally Mediated Malignancies

Despite major advances in therapies for several cancers, most patients with advanced cancer eventually succumb to the underlying malignancy. Many cancers carry considerable genomic complexity at diagnosis and acquire mechanisms of resistance to current therapies, including chemotherapy, targeted therapy, and immune therapies. Even the most successful cancer immune therapies, such as immune checkpoint inhibitors and adoptive transfer of engineered T cells, only benefit a subset of patients and are not amenable to easy application for the prevention of cancer, particularly in the developing world. In addition to the need to reduce human suffering and mortality from cancer, the increasing and unsustainable costs of cancer care also create an economic argument to reduce the cancer burden, even in rich nations (1). One such approach to prevention is vaccination, which has been highly effective against some pathogens. In the setting of virally mediated disease, evidence is emerging that preventive vaccines for reducing viral infections are also effective for preventing virally mediated cancers (2). The risk of chronic liver disease and hepatocellular carcinoma (HCC) following hepatitis B virus (HBV) infection is higher in children who acquire the infection before the age of 5 years (3). HBV infant vaccination programs have shown remarkable efficacy in the reduction of HCC incidence compared to non-vaccinated controls (3, 4). Vaccines against human papillomavirus (HPV) represent another success story in terms of protection from virus-induced malignancy (2, 5, 6). Two currently approved HPV vaccines provide protection not only against chronic infection with HPV types 16 and 18 but also against cervical intraepithelial neoplasia (CIN), adenocarcinoma *in situ*, and cervical cancer. Vaccines targeting E6 and E7 antigens from HPV 16 and 18 have also shown remarkable efficacy in mediating the regression of CIN lesions (2). For example, women with grade 3 vulvar lesions vaccinated with long peptides derived from these antigens experienced high rates of complete regression of these lesions (7). In the Papilloma Trial against Cancer in young Adults (PATRICIA trial), HPV vaccination led to complete protection from CIN as well as adenocarcinoma *in situ* lesions (5, 6). In view of its effects on precursor lesions, it is projected that HPV vaccination will lead to a major reduction in cervical cancer mortality in the next 20–30 years. One important lesson from this experience is that vaccines incorporating antigens that do not lead to regression of established cancers are still highly effective in preventing early lesions.

## Immune Surveillance and Editing: Insights From Mouse Models

Although it has been over 50 years since the initial evidence for immunity against carcinogen-induced tumors in mice was published, the concept that the immune system could mediate surveillance against tumors has now overcome initial skepticism (8). Several strains of immune-deficient mice have been shown to be deficient in immune surveillance in one form or another in models that include both carcinogen-induced and spontaneous cancers. Schreiber and colleagues proposed the term cancer immune editing, which incorporates three distinct phases: elimination, equilibrium, and escape (8). An important aspect of the equilibrium phase, as different from prior concepts of dormancy, is that the tumor is not really static but is likely engaged in ongoing interactions with the immune system leading to evolution (or editing) until there is escape from immune destruction (9). A deeper understanding of the equilibrium phase is particularly critical for translation to secondary cancer prevention in the clinic, as it resembles the premalignant or clinically silent phase preceding cancer.

## Host Response to Preneoplastic Lesions in Humans

Most studies of cancer immunity in humans have focused on patients with clinical cancer, which represents the escape phase. In this setting, the presence of immune infiltration within tumors has emerged as a strong predictor of outcome, in some cases more dominant than the clinical staging systems currently in place (10). Indeed, the presence of pre-existing tumor immunity forms the basis for the clinical success of immune checkpoint therapies (11). However, genomic studies have shown that many of the oncogenic mutations are acquired long before the clinical malignancy is manifest (12). Studies on such human precancer lesions are limited, as these lesions (e.g., colon polyps) are typically resected at the time of initial diagnosis. However, even in these settings, it has been shown that there are changes in adjacent “normal” mucosa that predict the risk of recurrence (13), thereby making a case for targeting these abnormal cells to reduce recurrence. The presence of immune infiltration has now been demonstrated in diverse preneoplastic states including intraductal papillary mucinous neoplasms (IPMNs) that precede pancreatic cancer (14, 15), oral leukoplakia as a precursor to oropharyngeal cancer (16), non-invasive bladder cancer (17), bronchial lesions preceding lung cancer (18–20), and ductal carcinoma *in situ* (DCIS) of the breast (21–24). One of the earliest examples of specific immune responses to human preneoplasia in the tumor microenvironment was in the setting of monoclonal gammopathy of undetermined significance (MGUS), which serves as a precursor to myeloma (MM) (25). In contrast to other cancers, tumor cells in MGUS cannot be surgically resected at initial diagnosis, and therefore it provides an important and unique model for studies on early response to preneoplastic lesions in humans (26). Notably, although MGUS lesions carry many of the genetic changes found in MM cells, only a small proportion go on to develop clinical malignancy (26, 27). Prior studies have shown that the immune system does recognize these lesions, and this leads to alterations in both innate and adaptive immune cells in the bone marrow (25, 28–31). Importantly, pre-existing T cell immunity was a strong predictor of reduced risk of progression to clinical myeloma in a large prospective clinical trial, with protective effects manifest across all major genetic subtypes of MGUS (32, 33). As is the case with precursor states to more common solid tumors, MGUS lesions are quite common and can be detected even with less sensitive methods in up to 3% of individuals over 50 years of age (26). It is important to note that while MGUS is not surgically resectable as in some other preneoplastic lesions, several aspects of the biology and genetics of these lesions resemble the more common solid tumor counterparts. For example, genome sequencing studies have shown that precursor and pre-invasive lesions in solid tumors carry many of the genomic alterations found in their clinically malignant counterparts, and this is true in the setting of MGUS as well (27, 34).

Chronic immune responses can lead to T cell dysfunction or exhaustion (35). As the premalignant phase of cancer is immunogenic and lasts much longer than the malignant phase itself (typically several years), an important question arises—how does the host maintain such a chronic immune response? In mouse models of chronic viral infections such as lymphocytic choriomeningitis virus (LCMV), the maintenance of chronic immune responses and the prevention of the attrition of exhausted T cells depend on the presence of a subpopulation of stem-like T cells (36–38). Loss of this subset leads to attrition of the immune cells and loss of immunity in these models (36). Similar biology may also be operative in the setting of premalignancy. Utilizing complementary single-cell technologies, T cells infiltrating MGUS lesions were found to be less differentiated than those seen in MM (39). These cells were also enriched for TCF1/7+ memory T cells as well as those with tissue-resident phenotypes (39). Therefore, the hierarchy of T cell exhaustion seems to be established early in the setting of cancer development. Another insight from these studies is that changes in innate immunity as well as in the myeloid compartment also occur early (15, 30, 39). Early emergence of suppressive myeloid populations may be an important obstacle to immune-based prevention targeting these lesions (15, 40). An important challenge in terms of studying the biology of host response to human preneoplasia relates to the limitations of existing models in terms of permitting the growth of human preneoplastic cells *in vivo*. Recent advances with humanized models do permit the growth of human premalignant MGUS cells *in vivo* (41) and may provide a useful tool for probing these questions.

## Antigenic Targets For Cancer Prevention

Ideally, an antigenic target for a preventive vaccine would be highly tumor-specific, essential for tumor biology, expressed by the entire clone (or clonogenic progenitors), and capable of eliciting an immune response of sufficient potency to mediate protection. Advances in cancer genetics have shown that the genomic complexity of cancer is established early, even during the premalignant stages and that the tumor in each patient has a distinct set of genomic alterations and oncogenic mutations that yield neoantigenic targets (42). While this suggests the need to consider personalized approaches such as those targeting mutation-associated neoantigens (MANA) to prevent cancer (discussed later), strategies that target non-mutated tumor-associated antigens shared between tumors present fewer logistical challenges and are more amenable to clinical testing. One such antigen is MUC1, which is immunogenic in several human preneoplastic states and has therefore emerged as an attractive target for such preventive approaches (43). For example, intraductal papillary mucinous neoplasms (IPMNs) as precursors to pancreatic cancer express a hypo-glycosylated form of MUC1 and develop IgG antibodies against this antigen (15). Heavy smokers with preneoplastic lung lesions were shown to develop IgG antibodies against cyclin-B1 (44). HER2 is overexpressed on tumor cells in ductal carcinoma *in situ* and leads to the induction of immune responses in this setting (22, 45). Progression to invasive breast cancer is associated with a decline in these responses, setting the stage for targeting this antigen in the context of preventive vaccines (22, 45). The efficacy of vaccines against these antigens has also been demonstrated in murine models of breast cancer (46). An antigen screen for immune-reactivity in MGUS suggested that shared antigenic targets of host response in MGUS may differ from the malignant counterpart, myeloma (28). Specifically, the top antigenic targets in MGUS were genes such as SOX2 that are known to play a role in the biology of embryonal stem cells and are enriched on clonogenic progenitors (28, 47). In murine models, vaccines targeting early-stage antigens were more effective than vaccines targeting antigens expressed later in the course of the cancer (48). The presence of a T cell response against SOX2 emerged as an independent predictor of reduced risk of malignancy in MGUS in a large prospective study (32). Recent studies have also shown that OCT4, another embryonal stem cell-associated gene, can be immunogenic in humans (49). T cell responses against these antigens have also been observed in the setting of tumor regressions in the setting of checkpoint blockade, chimeric-antigen-receptor (CAR)-T cells, and chemotherapy of highly curable germ cell tumors (49–51). Further studies are needed to better understand whether immune targeting of stemness pathways in preneoplastic lesions can be clinically exploited for immune prevention (52, 53).

## Mutation-Associated Neoantigens as Targets For Prevention

As much of the antitumor-response in preneoplastic lesions seems to be specific to an individual lesion (25), mutation-associated neoantigens (MANA) may be an important target for T cell response-targeting for cancer prevention. The importance of the T cell response against MANA has been demonstrated in mouse models and can impact the evolution of tumors during the equilibrium phase (54, 55). Serial analyses of human cancer have also provided evidence of immune-mediated regulation of cancer evolution, including that involving neoantigens (55, 56). However, whether T cells against neoantigens are essential for effective cancer prevention in the clinic remains to be established. Several studies have tried to vaccinate cancer patients against neoantigens in order to elicit MANA-specific T cells *in vivo* (42). While these studies have shown the feasibility of eliciting such responses, they seem to be of low frequency compared to immune responses following viral infections, and whether they mediate clinically meaningful anti-tumor effects remains to be established. It should be noted that as the genomic makeup or tissue of origin of cancers cannot currently be predicted before they develop, most of the efforts toward cancer prevention are only feasible as secondary cancer prevention, such as in patients with preneoplastic states. Primary cancer prevention is, however, potentially attractive in the case of hereditary cancer syndromes with defined patterns of organ-specific cancer, such as patients with Lynch syndrome.

## Insights From Vaccines in Chronic Viral Infections

If preventive vaccines in cancer can realistically only be tested in the setting of pre-existing preneoplasia at present, then some of the lessons learned from mouse models and human studies of chronic viral infections such as human immune deficiency virus (HIV), hepatitis C virus (HCV), and hepatitis B virus (HBV) are worth considering. In chronic viral infections, the T cells target non-self-epitopes, similar to their response against neoantigens. Vaccines generally lead to poor T cell expansion in the case of chronic infection with the clone 13 strain of lymphocytic choriomeningitis virus, which leads to chronic viral infection (57). In the case of simian-immunodeficiency-infected primates, prior reduction of viral load with anti-retroviral therapy was required in order to elicit strong T cell responses to gag antigens (58). Peptide and viral vaccines against hepatitis B and C have led to only mild increases in T cells against target antigens in infected patients, although the ability to elicit T cells in uninfected individuals was much greater (59–61). Chronic exposure to the virus also leads to a loss or a reduction in the loss or deletion of T cells with the highest affinity to the antigen. For example, in chronic gamma-herpesvirus infection, high-affinity clones mediate early robust expansion but undergo attrition, while intermediate/low-affinity clones are maintained longer (62). Similar observations have been made in human HIV infection (63). These considerations raise the possibility that T cell responses, even to neoantigens, may not be as impressive as currently hoped if applied late in the course of preneoplasia.

## Lessons From Therapeutic Vaccination in Cancer

The discovery of the T cell response to tumor-associated antigens, beginning with the MAGE family (64), not only provided the foundation for the field of cancer immunology but also led to studies of therapeutic vaccination. Several strategies have been utilized for inducing immunity to tumor-associated antigens. These include injection of peptides with adjuvants, DNA vaccines, viral vectors, dendritic cell vaccines, and prime-boost approaches (65). Prime-boost approaches have also commonly been utilized in the case of chronic viral infections. With increasing appreciation of the importance of MANA, several of these strategies are currently being applied to try to elicit immunity to neoantigens in the clinic (42). However, many of the initial studies focused on patients with clinical malignancy but often lacking measurable disease, and the clinical efficacy of such approaches remains to be established (42). The vaccination field was greatly aided by the discovery of dendritic cells (DCs) as critical antigen-presenting cells and led to several studies targeting mature DCs (66–68). However, while monocyte-derived DCs led to T cell responses in several patients, these studies led to tumor regressions in only a small proportion of patients, although some of these responses have been long-lasting (67, 69). Only one of the DC vaccines, Sipuleucel-T, has to date led to improved survival in the setting of cancer (70). It is important to note that the initial studies did not target the immune-suppressive pathways, including immune checkpoints and regulatory T cells. More recent studies have successfully targeted human DCs *in situ*, which is more amenable to larger-scale clinical trials (71). However, these studies were also conducted without addressing immune-suppressive factors in the tumor bed. Vaccine-based studies exploiting the biology of human DC subsets, and in particular those with enhanced potential for cross-presentation, have not yet been carried out, although evidence for the feasibility of targeting these subsets is emerging (72, 73). Strategies that target DCs directly *in situ* may also be preferable to those that target DCs *ex vivo* because the former may allow targeting of naturally occurring DCs in greater numbers compared to those limited by the effect of *in vitro* culture (71). In this regard, specific targeting of DC subsets *in situ* remains an unmet need. It is possible that combinatorial targeting of defined DC subsets may be essential for robust immunity (73, 74). An important desired goal of vaccines is to elicit T cells that mediate long-term protection (75). It has been suggested, for example, that vaccines that elicit tissue-resident memory T cells may be needed to mediate protective immunity (76). Studies with yellow fever vaccine, one of the most effective vaccines in humans, have provided important insights into the properties of long-term protective immunity, involving the induction of a broad immune response and the generation of long-lasting memory T cells (77, 78). It remains to be demonstrated whether T cells with similar properties can be elicited in the context of vaccination against tumor antigens.

## Diversity of Preneoplastic Lesions—Do We Need to Link Immune Prevention With Early Detection?

**Figure 1:**
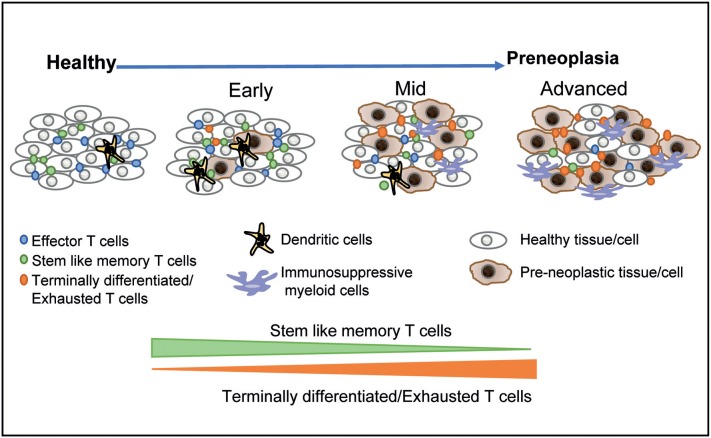
Immunological diversity and evolution of precursor states. The application of single-cell technologies to studying precursor states has shown that the earliest lesions are associated with changes in the immune microenvironment. The hierarchy of T cell exhaustion is established early and is associated with a relative decline in stem-like and resident memory T cells over time. More advanced lesions are associated with infiltration by more immune-suppressive myeloid populations. These data suggest that immune prevention through vaccination may be most effective for earlier lesions, with more advanced lesions requiring combination strategies.

It is now well-appreciated that preneoplastic lesions can exhibit significant diversity. At the clinical level, this includes features such as size, dysplasia, and genomic changes in preneoplastic cells that confer an increased risk of malignant transformation. However, these lesions may also differ considerably in terms of the nature of the host immune response. As discussed earlier, many of the oncogenic mutations found in cancer cells originate in the precursor phase. The initial studies describing the presence of expanded hematopoietic clones carrying genomic mutations have now been extended to clones of cells with somatic mutations within normal tissues in otherwise healthy individuals (79, 80). The long natural history of these lesions, typically spanning several years, implies (although it is not proven) that the immune system has already undergone chronic exposure to these antigens. The application of single-cell technologies to study the immunology of these lesions has illustrated the diversity of human preneoplastic states, wherein the immune response evolves over time (Figure 1) (18, 19, 39). As discussed earlier, the persistence of exhausted T cells in models of chronic viral infection depends on a subset of T cells that exhibit more stem-like features (36). Recent studies in MGUS patients have shown that similar hierarchies of T cell exhaustion that are responsible for maintaining chronic T cell responses are established early during carcinogenesis (39). Advanced lesions also carry greater dysfunction of innate cells including NK cells, innate lymphoid cells, and altered polarization of myeloid cells (30, 39). Changes in the myeloid compartment may therefore be an important driver of the malignant phenotype and the loss of immune control (15, 39, 40). Strategies that target innate immunity may therefore also be explored for cancer prevention (81). The concept that precursor lesions are not immunologically silent suggests that strategies that overcome immune checkpoints may also be effective in these patients. While current strategies for checkpoint blockade do carry the risk of adverse autoimmune events that may be unacceptable for this patient population (82), advances in preventing such complications (83) may make it more feasible to pursue checkpoint blockade to target high-risk precursor lesions.

The concept that the immunologic evolution of the tumor microenvironment begins early also has important implications for the timing of immune prevention. It may be desirable to target lesions that still have high levels of stem-like and tissue-resident T cells and low levels of immune-suppressive myeloid cells in order to achieve a durable response to immune-mediated prevention. This, in turn, may require that strategies that pursue immune prevention are directly linked to early detection before the adverse aspects of the preneoplastic immune microenvironment are fully established. Alternatively, combination approaches (such as are being pursued in the context of therapeutic manipulation of immunity in established cancers) may be required for immunologically altering the natural history of more advanced preneoplastic lesions. Traditionally, the rationale for early detection in cancer has been limited to enhancing the potential for the surgical resection of the lesion, presumably with curative intent (84). Here we suggest that even in a setting wherein surgical resection is not feasible (e.g., hematologic premalignancies) or is clinically not indicated, early detection may be essential for achieving a window of opportunity for effective immune prevention.

## Clinical Studies of Immunoprevention

In contrast to the large body of literature evaluating therapeutic vaccination in cancer, data about preventive vaccination, particularly for non-viral vaccines, are limited. One of the antigens evaluated in more advanced studies is the tumor-associated antigen MUC-1. The safety and immunogenicity of a MUC-1 peptide vaccine have been demonstrated in initial clinical studies (85). While colon polyps are typically resected at diagnosis, the rationale for vaccination in this setting is based on reducing the recurrence of polyps. In the initial studies, the immunogenicity of the vaccine was impaired in patients with elevated myeloid suppressor cells, suggesting that vaccination in the earlier stages of preneoplasia should be considered, as discussed earlier (85). Nonetheless, MUC-1 vaccination is currently being tested in the context of a phase III trial. Instillation of Bacillus Calmette Guerin (BCG) has been shown to mediate the regression of *in situ* bladder cancer lesions but is ineffective in the setting of more advanced muscle-invasive lesions (17). Vaccination in the neoadjuvant setting has been trialed to evaluate the induction and anti-tumor effects of vaccination for preneoplasia. Vaccination of women with DCIS of the breast with dendritic cell vaccines presenting Her2-derived peptides led to the induction of immunity and provided some early evidence of antitumor effects, with a reduction in DCIS lesion seen in some patients at surgery (45). Preneoplastic lesions that cannot be resected (as is the case with MGUS, a precursor to myeloma) represent an attractive model for establishing the principles of immunomodulation for the prevention of human cancer. In a recent study, patients with smoldering myeloma (an intermediate preneoplastic stage between MGUS and myeloma) were randomly assigned to either observation alone as the standard of care or administration of single-agent lenalidomide, an immunomodulatory drug (86). Lenalidomide led to a significant prolongation of progression-free survival compared to observation, with a nearly 70% reduction in the risk of clinical malignancy (86). These data provide an example of successful immune-modulation-based interception of human cancer (87), in this case utilizing an oral therapy that would otherwise be inadequate as a single agent in the setting of established cancer. These findings may not only change clinical practice for the subset of patients at highest risk of clinical progression; they also set the standard for future studies testing immune-based prevention in MM.

## Challenges and Barriers to Preventive Vaccines and Other Approaches

In spite of an improved understanding of the immunology of precursor states, there are several potential challenges to preventive vaccination of cancer, even when targeting preneoplastic lesions (88). At present, it is not feasible to accurately predict which specific antigen (or combination thereof) will serve as a rejection antigen or effectively prevent cancer in an individual patient. While peptide-based strategies have been employed to target both shared antigens and neoantigens, several variables, such as the choice of peptides and their immunogenicity, clearance, and expense may impact the clinical efficacy and application of peptide-based vaccines. Targeting a limited set of antigens also carries the potential for antigen-loss variants as a mechanism for immune escape. Antigen-loss has been shown to be a potential mechanism of tumor immune escape in murine models (54, 89). However, the degree to which this occurs in the setting of preventive vaccination in the clinic remains to be established. One potential strategy may be to target “trunk” mutations or genes essential for a malignant phenotype, but this has, to date, proven challenging in the clinic, and several of the trunk mutations may not be immunogenic (42). Other barriers that limit therapeutic cancer vaccines may also apply to preventive vaccination, particularly if the latter is approached in the setting of more advanced precursor lesions. These include intra-tumoral heterogeneity, stem-like features of tumor cells or even putative cancer stem cells, and other immune-suppressive features in the tumor microenvironment (90). If true, this would imply that preventive vaccination would also need to use combination approaches as is currently being explored in the setting of established cancer. As discussed previously, these considerations further reinforce the need to link immune prevention to early detection, and perhaps even before clinically meaningful preneoplastic lesions are manifest. In addition to vaccines, other strategies such as T-cell redirection (e.g., bispecific antibodies) and other immunomodulatory antibodies are being considered for immune-based interception. Recent success with lenalidomide in the prevention of myeloma, as discussed earlier, may encourage such studies. However, given the cost and potential toxicity, it would be important to limit such approaches to patients at highest risk and with careful attention to long-term effects.

## Summary

In the preceding sections, we have discussed the emerging evidence in support of immunological approaches to preventing cancer. In contrast to therapeutic vaccination, these are still very early days for clinical or even translational studies testing these hypotheses. However, advances in cancer genetics and recent successes in cancer immunotherapy have begun to set the blueprint for strategies to harness tumor immunity to prevent cancer. It is now being appreciated that clonal expansions of cells carrying potentially oncogenic mutations are common in healthy tissues (80). As the biological and immunological principles underlying these strategies are being established, careful clinical investigation will be required to move the field forward. One of the challenges that makes cancer a formidable foe is its ability to adapt and evolve, as is also the case with pathogens. Therefore, the immune system, with its capacity to adapt, evolve, and persist, may be our best defense against cancer, as is already evident from its success in preventing pathogens (91). Planned investments in defining the landscape of precursor states to human cancer should go a long way toward helping us achieve these goals (12, 92).

## Author Contributions

All authors listed have made a substantial, direct and intellectual contribution to the work, and approved it for publication.

### Conflict of Interest

The authors declare that the research was conducted in the absence of any commercial or financial relationships that could be construed as a potential conflict of interest.
